# Views of Dr. Addison

**Published:** 1920-05-29

**Authors:** 


### CO-ORDINATION OF LONDON HEALTH SERVICES.

### Views of Dr. Addison.

THERE is a likelihood that the proposals of the London County Council for the co-ordination of London health services and the formation of a Central Council of London Hospitals will materialise in due time inasmuch as the proposals have been favourably received by Dr. Addison. In support of its proposals the L.C.C. sent a deputation to the Ministry of Health. The proceedings were private, but the County Council has now issued an official report which gives the subjoined reply by Dr. Addison, from which we have made the essential abstracts:—

I think that in any case it is obvious that the problem of London is to a great extent *sui generis*. Clearly in any scheme which is proposed, the London question must be specially dealt with. . . . Various technical points were raised which you will not expect me to-day to deal with in reply, but one thing I think is obvious. It is perfectly clear that a great deal of the present muddle has arisen from the needless multiplication of authorities, and also that there are certain services which are administratively local, and others which clearly are not. But in regard to many services it is essential that you should have an authority which is competent to deal with them over a considerable area, and the more highly specialised they become the wider generally must be the range of authority of whoever deals with it. Of course in these days, when things in some directions are becoming more and more specialised, it may well happen—I think not in the case of London where you have everything to hand, but in other places—that you might have some services for which the county unit might not be big enough, say, the provision of laboratory services, highly specialised services of different kinds. So it is plain that we have to contemplate in respect of many services a health authority competent to handle over a large field, competent to set up the necessary skilled and other services, competent to survey the whole field in respect of them, to prepare schemes and whatever machinery we may adopt for securing that we get good administration in the area. You have to have a sort of general staff in health matters with bigger units of health government, and until you get a general staff in these matters you cannot possibly get comprehensive and consistent scientific dealing with health services. That goes without saying, and it is obvious it applies particularly to the metropolitan area. . . . I think that in some directions, as the responsibility of big authorities becomes greater and greater, you are bound to call to your aid persons with special knowledge and experience who do not care to run the risk of being unsuccessful in an election. Therefore, I welcome that suggestion, whilst that does not in any way detract from the authority itself having the final voice, having, of course, the financial responsibility. I do not know to what extent you have been able to come to any understanding in respect of the marginal services with the borough councils. If you have got complete agreement, or can get it, you will have achieved a great success. I would not anyhow be very sanguine on that, but at the same time I do think it would be useful, it would certainly be helpful to me if you could talk it over, and see whether you could devise some means for discussing these matters without prejudice with representatives of the boroughs with the view of endeavouring to reduce the number of points of disagreement as much as may be consistent with there being an adequate and proper scheme for the area as a whole. . . .

In one thing I am in entire agreement with what you said, that we must have a system whereby an adequate share of the financial responsibility must fall upon the authorities administering the services, otherwise we have no check on extravagance and other things. As to that, I am quite sure we are in agreement.

You then raised the general question of the London hospitals. There is a good deal to say for that suggestion that you have put forward. At the present time I may say that we have discussed the position with the leading representatives of some of the bigger voluntary hospitals, and we shall be meeting them again, particularly with regard to their present financial difficulties. But it is inconceivable that any system of health authority could be set up which did not include the hospital system. Of course, anyhow, we have to remember that 50 per cent. of the beds, notwithstanding the invaluable and highly skilled work of the London hospitals, 50 per cent. of the beds in the country generally, I do not know how it is in the London area, are in the Poor-Law infirmaries, so that it seems to me you cannot split up the hospital supervision. I am only talking in general terms, you do not expect me to do more than that.

All I have now to say is that your main principles, or some of them at all events, governing your recommendations, I think, are unassailable, and anyone who has studied this question would recognise that. We shall have no doubt abundant opportunities in time to come of discussing these things in detail, and I welcome your offer of friendly co-operation.

In view of the satisfactory nature of Dr. Addison's reply the L.C.C. has arranged for further conferences with the Borough Councils.

STRATFORD-ON-AVON.—The Governors of the General Hospital have approved a scheme for enlarging the institution by increasing the ward area from thirty-one to fifty-one beds, by building a new out-patient department, and by improving the operating-theatre and service department, at a cost of £26,250.

